# The relationship between masticatory ability, age, and dental and prosthodontic status in an institutionalized elderly dentate population in Qingdao, China

**DOI:** 10.1007/s00784-018-2477-z

**Published:** 2018-05-07

**Authors:** Qian Zhang, Dick J. Witter, Ewald M. Bronkhorst, Nico H. J. Creugers

**Affiliations:** 1 Department of Oral Function and Prosthetic Dentistry, College of Dental Science, Radboud University Nijmegen Medical Centre, Philips van Leydenlaan 25, 6525 EX Nijmegen, The Netherlands; 2 Department of Preventive and Restorative Dentistry, College of Dental Science, Radboud University Nijmegen Medical Centre, Philips van Leydenlaan 25, 6525 EX Nijmegen, The Netherlands

**Keywords:** Masticatory ability, Institutionalized elderly, Dental status, Prosthodontic status

## Abstract

**Objectives:**

To identify relationships between masticatory ability and age, and dental and prosthodontic status amongst an institutionalized elderly dentate population in China.

**Materials and methods:**

A sample of 512 elders living in eight nursing homes in Qingdao was categorized based on a hierarchical dental functional classification system with and without tooth replacements. Masticatory disability scores (MDSs) were analyzed using multiple regression models with only age, and age and dentition variables for participants having ≥ 10 natural and those having < 10 natural teeth in each jaw.

**Results:**

Overall, associations between MDS and age, number of teeth, and number of teeth replaced by dental prostheses were identified. For participants having ≥ 10 natural teeth in each jaw, no significant associations between MDS and age and dental and prosthodontic status were found. Participants having < 10 natural teeth in each jaw had higher MDS (increasing chewing difficulties) at higher ages*.* However, when “premolar region sufficient” and “molar region sufficient” were included, MDS was not associated with age, but with these dentition variables. For participants having ≥ 10 teeth including prosthodontically replaced teeth in each jaw, age was the only variable associated with MDS. For participants having < 10 teeth including teeth replaced in each jaw, the significant factor was “premolar region sufficient.” Overall, lower MDS was associated with increasing number of teeth, as well as with increasing number of teeth replaced by dental prostheses.

**Conclusions:**

In this population of institutionalized dentate elderly, masticatory ability was significantly associated with dental and prosthodontic status.

**Clinical relevance:**

For institutionalized elderly, having less than ten natural teeth in each jaw is associated with chewing problems. Most important dentition factor is the presence of three to four premolar pairs. Teeth added by partial removable dental prostheses compensate impaired masticatory ability due to tooth loss for 50% compared to natural teeth.

## Introduction

Masticatory ability is an important determinant of oral wellbeing, particularly for elderly individuals [1, 2]. Chinese studies report a high risk of eating difficulties for older people with fewer than 20 teeth [3, 4]. A study amongst older Americans found that people with severe tooth loss (≤ 10 remaining teeth) were less likely to meet the dietary recommendations of the Healthy Eating Index 2005 than those with light to moderate tooth loss (≥ 11 remaining teeth) [5, 6]. The relationship between dental status and dietary intake was also confirmed in a longitudinal study amongst older Japanese [7]. Moreover, chewing difficulties in elderly people are associated not only with a risk for nutritional problems but also with impaired cognitive functions [8–11].

Masticatory function has been associated with number of teeth and occluding pairs, along with other age-related factors, such as muscle strength, saliva flow, and the use of medication [12, 13]. Longitudinal studies amongst community-dwelling elderly populations have indicated decreasing masticatory ability with increasing age [14, 15]. The relationship between masticatory ability and dental and prosthodontic status has been the subject of numerous cross-sectional studies, with the majority of studies reporting a strong association [4, 16–18]. Apart from the number of natural teeth, the number of teeth replaced can also contribute to masticatory function. While sensory and motor feedback to the central nervous system from dental prostheses is impaired, it is considered that tooth-supported fixed dental prostheses (FDPs) can compensate for this impairment and people with FDPs can obtain a masticatory function close to that of natural teeth [19]. Partial removable dental prostheses (PRDPs) have been reported to compensate only partially; for example, a systematic review demonstrated that distal-extension RDP in shortened dental arches provided only 50% of the masticatory efficiency of complete dental arches [20].

In China, demographic changes have brought about a nationwide shift from traditional family care for elders to institutionalized care [21]. Currently, approximately 1.5% of older people live in nursing homes and apartments for the elderly, but it is expected that this figure will increase in the coming years [22]. A 2016 study amongst institutionalized elderly in China showed that a large proportion of these people had a dental status considered non-functional [23]. To our knowledge, so far, the relationship between dental status, masticatory function, and age in institutionalized elderly has been investigated only rarely. One such study reported a positive association between number of teeth and chewing ability; however, this study was conducted amongst a group of institutionalized xerostomia patients [24]. In a Japanese study amongst nursing home residents, masticatory ability was associated with general health, number of teeth, and bite force [25]. Another study, amongst Korean community-dwelling and institutionalized elderly, reported no significant differences amongst these groups in the relationships between number of teeth, masticatory ability, and oral health-related quality of life [26]. Yet, for institutionalized Chinese elders, no data are available that link masticatory ability and dental status.

The aim of this study was to investigate masticatory ability in an institutionalized elderly dentate population in China. It was hypothesized that self-assessed masticatory ability would be associated with age and with dental and prosthodontic status.

## Materials and methods

### Participants

The present study was conducted in Qingdao, a city with approximately three million inhabitants that is located on the east coast of Shandong Province, Eastern China. A purposive sample of eight elderly care homes (varying from 33 to 359 residents; total number of residents = 1226) in different districts in Qingdao was selected on the basis of accessibility and convenience. Information on the purpose and procedures of the study was provided to the management of the care homes and their residents. The study aimed to include a total of 500 participants.

All residents were visited room by room and invited to participate in the investigation. A total of 512 people (42% of the total population of the visited elderly care homes) who were capable of communication and presented no indication of cognitive impairment and no life-threatening condition agreed to participate. The number of participants per care home ranged from 7 (21% of the residents of that particular care home) to 171 (86%); 66% of the participants were females. The study was carried out in compliance with the Helsinki Declaration. Prior to the start of the study, the ethics committee of the Affiliated Hospital of Medical College, Qingdao University, approved the study protocol.

### Questionnaire

Participants were asked to complete a structured questionnaire that had been used previously in an epidemiological study in Qingdao [27]. The questionnaire included questions about whether the participant was able to chew eight different foods commonly eaten by Chinese people: four foods considered soft (cooked rice, steamed bread, *shaobing* (Chinese-style baked flour roll), and cooked meat) and four considered hard (raw vegetables, raw carrots, apples, and nuts). Perceived difficulty of chewing was scored for each food as follows: score = 1: very easy to chew; score = 2: minor problems with chewing, got used to it; score = 3: minor problems, cannot get used to it; score = 4: difficult to chew, not avoiding this food; score = 5: very difficult to chew, not avoiding this food; score = 6: very difficult to chew, avoiding this food. If participants recorded “7 = not avoiding this food, but never eaten it,” this score was excluded from the analyses [28].

All participants understood Mandarin; however, some participants were not able to complete the questionnaire by themselves (e.g., because of illiteracy or visual impairment), and these were helped by an assistant who read aloud the questions and recorded the answers. After completion, each questionnaire was checked for unrecorded items and, if applicable, participants were requested to complete those items.

### Clinical examination

In accordance with the study protocol, verbal informed consent was obtained from each participant before they entered the study. Two calibrated dentists trained by an experienced researcher performed the oral examination following the procedures and diagnostic criteria recommended by the World Health Organization [29]. Inter-observer agreements amongst the experienced researcher and the two dentists on these variables were excellent (all kappas ≥ 0.8). In the present study, of all variables recorded, only the presence of teeth (including third molars), tooth type, number and location of posterior occluding pairs (pairs of opposing natural premolar and/or molar teeth), and tooth replacements were considered. Retained roots were regarded as non-functional and as candidates for replacement and, therefore, considered missing teeth. Replaced teeth were recorded as missing teeth replaced by FDPs or PRDPs. Posterior occluding pairs (POPs) reconstructed by FDP and/or PRDP were considered as reconstructed posterior occluding pairs (R-POPs).

### Data analysis

SPSS version 22.0 (SPSS Inc., Chicago, IL, USA) was used for data analyses. Participants that were edentulous in one or both jaws were excluded from the analyses. Dentitions were classified on the basis of the multi-level hierarchical dental functional classification system that had been used previously in epidemiological studies in different countries [28, 30–32], in which the criteria applied at the levels are based on conditions that reflect oral functionality (Table 1). The criteria in this system are based on number and type of teeth present and number of posterior occluding pairs. Participants were classified in two ways in the hierarchical dental functional classification. First, participants were classified on the basis of the presence of ≥ 10 or < 10 natural teeth in each jaw only. Next, in order to evaluate the effect of tooth replacements on masticatory ability, they were reclassified on the basis of their dental status ≥ 10 teeth or < 10 teeth in each jaw, including natural teeth plus teeth replaced by FDP and/or PRDP. The scores for eight foods were transferred to the masticatory disability score (MDS), which is the average score for the eight combined foods. Additionally, the scores for the four soft and the four hard foods were averaged into an MDS for soft and an MDS for hard foods.

**Table 1:** Levels and criteria for dichotomization in the multi-level hierarchical dental functional classification system (HDFC)

Level	Meeting criterion		Dichotomy
	Yes	No	
I. Dentition level	≥ 1 tooth present in each jaw	Edentulous jaw(s)	≥ 1 tooth versus no teeth
II. Jaw level	≥ 10 teeth in both maxilla and mandible	< 10 teeth in maxilla or mandible	≥ 10 teeth versus < 10 teeth
III. Anterior level	All 12 anterior teeth present	< 12 anterior teeth	Complete versus incomplete
IV. Premolar level	3 or 4 occluding pairs of premolars	≤ 2 occluding pairs of premolars	“Sufficient” versus “impaired”
V. Molar level	≥ 1 occluding pairs of molars at both left and right sides of the dentition	No occluding pairs of molars at left or right side of the dentition	“Sufficient” versus “impaired”

For the participants within these groups, mean numbers of natural teeth, of teeth replaced by FDPs and/or PRDPs, posterior occluding pairs and reconstructed posterior occluding pairs, and mean MDS were calculated. Next, the MDS for different ages and different dental and prosthodontic statuses was analyzed in regression models. In the first model, “age” was the independent variable; in the second model, “age” and “anterior region complete”; in the third model, “age” and “premolar region sufficient”; and in the fourth model, “age,” “premolar region sufficient,” and “molar region sufficient” were the independent variables. Finally, the effects on MDS (dependent variable) of age per year, each natural tooth present, each “tooth” added by FDP, and each “tooth” added by PRDP (independent variables) were analyzed in a regression model as well.

## Results

Of the 512 participants, 360 (70%) reported their perceived general health as being fair to excellent, while 152 (30%) reported their health as being poor. Seventy-five percent (*n* = 384) of the participants were dentate in both jaws; 25% (*n* = 128) were edentulous in one or both jaws, of which 58 (11% of the total sample) were completely edentulous. More than half (62%) of the participants were aged 80 and over (Table 2).

**Table 2:** Number (%) of participants, % female participants, and number (%) of participants dentate in each jaw according to age groups

Age groups	Participants	% female	Dentate in each jaw
60–69	47 (9)	66	44 (94)
70–79	150 (29)	70	131 (87)
≥ 80	315 (62)	64	209 (66)
Total	512 (100)	66	384 (75)

### Masticatory ability and dental status based on natural teeth only

The mean number of natural teeth for participants having ≥ 10 teeth in each jaw was 26.27 ± 2.85 with 6.18 ± 1.96 posterior occluding pairs (Table 3). For this group, mean MDS was 1.79 ± 1.20. Participants having < 10 teeth in each jaw had a mean number of natural teeth of 13.37 ± 5.52, with 1.05 ± 1.45 posterior occluding pairs. Mean MDS was 2.94 ± 1.50, which indicates more chewing difficulties for participants having < 10 teeth in each jaw.

**Table 3:** Mean (SD) age, mean numbers (SD) of teeth and posterior occluding pairs, and masticatory disability scores for participants having ≥ 10 teeth or having < 10 teeth in each jaw, classified on the basis of natural teeth only and of natural plus replaced teeth

	Classified by natural teeth (*n* = 384)		Classified by natural teeth and teeth replaced (*n* = 384)	
	≥ 10 teeth in each jaw (*n* = 182)	< 10 teeth in each jaw (*n* = 202)	≥ 10 teeth in each jaw (*n* = 291)	< 10 teeth in each jaw (*n* = 93)
Age	77.07 (8.11)	81.25 (6.68)	78.56 (7.70)	81.48 (7.18)
Natural teeth	26.27 (2.85)	13.37 (5.52)	21.53 (7.38)	13.10 (5.45)
“Teeth” added by:				
FDP	0.62 (1.11)	1.09 (2.14)	0.95 (1.83)	0.61 (1.40)
PRDP	0.51 (1.56)	6.89 (7.76)	4.77 (7.12)	1.03 (2.90)
FDP/PRDP	1.13 (1.86)	7.98 (7.70)	5.72 (7.14)	1.65 (3.41)
Natural + replaced teeth	27.40 (2.60)	21.35 (7.45)	27.25 (2.33)	14.74 (5.96)
Natural POPs	6.18 (1.96)	1.05 (1.45)	4.30 (3.03)	0.90 (1.32)
Premolar region:				
POPs	3.36 (0.87)	0.67 (1.04)	2.35 (1.60)	0.69 (1.07)
POPs + R-POPs	3.62 (0.75)	2.38 (1.66)	3.62 (0.74)	0.90 (1.16)
Molar region:				
POPs	2.82 (1.54)	0.38 (0.80)	1.96 (1.74)	0.22 (0.62)
POPs + R-POPs	3.28 (1.45)	1.96 (1.76)	3.30 (1.32)	0.34 (0.73)
Total POPs + R-POPs	6.90 (1.84)	4.33 (3.22)	6.92 (1.72)	1.25 (1.49)
MDS soft	1.64 (1.04)	2.46 (1.34)	1.81 (1.11)	2.88 (1.42)
MDS hard	1.95 (1.47)	3.42 (1.87)	2.30 (1.63)	4.06 (1.83)
MDS all foods	1.79 (1.20)	2.94 (1.50)	2.05 (1.31)	3.47 (1.47)

*FDP*, fixed dental prosthesis; *PRDP*, partial removable dental prosthesis; *POP*, posterior occluding pair; *R-POP*, reconstructed posterior occluding pair; *MDS*, masticatory disability score

For participants having ≥ 10 teeth in each jaw, no significant associations were found between MDS and age, and age and dentition variables (Table 4: models 1 to 4). However, for participants having < 10 teeth in each jaw, the multiple regression analysis in the first model revealed an association between MDS and age (model 1: *P* = 0.023; *R*^2^ = 0.026); these participants reported more chewing difficulties at higher ages. The second model, in which the variable “anterior region complete” was added, showed an almost identical association for age (*P* = 0.030), but no significant effect for this dentition variable (model 2: *R*^2^ = 0.033). When the dentition variable “premolar region sufficient” was included, instead of “anterior region complete” (model 3: *R*^2^ = 0.076), MDS was not associated with age, but was negatively associated with “premolar region sufficient” (*P* = 0.001). In the model that included age and the variables “premolar region sufficient” and “molar region sufficient,” MDS was significantly associated with the status of both the premolar and molar regions: participants with “premolar region sufficient” and those with “molar region sufficient” reported fewer chewing difficulties (model 4: *P* = 0.008 and *P* = 0.020 respectively; *R*^2^ = 0.101).

**Table 4:** Multiple regression models for assessing associations between masticatory disability score (MDS) and age (model 1), and between MDS and age and dentition variables “anterior region complete” (model 2), “premolar region sufficient”(model 3), and “premolar region sufficient” plus “molar region sufficient” (model 4) for participants having ≥ 10 teeth in each jaw and those with < 10 teeth in each jaw, classified on the basis of natural teeth only and on the basis of natural teeth plus teeth replaced (*n* = 384)

	Classified by natural teeth						Classified by natural teeth + teeth replaced by FDP or PRDP or both					
	≥ 10 teeth in each jaw (*n* = 182)			< 10 teeth in each jaw (*n* = 202)			≥ 10 teeth in each jaw (*n* = 291)			< 10 teeth in each jaw (*n* = 93)		
	Effect	*P* value	95% CI*	Effect	*P* value	95% CI*	Effect	*P* value	95% CI*	Effect	*P value*	95% CI*
Model 1												
Age^a^	0.010	0.350	[− 0.011 … 0.032]	0.036	0.023	[0.005… 0.067]	0.029	0.004	[0.010 … 0.048]	0.024	0.258	[− 0.018 … 0.066]
	*R*^*2*^ = 0.005			*R*^2^ = 0.026			*R*^2^ = 0.029			*R*^2^ = 0.014		
Model 2												
Age^a^	0.010	0.364	[− 0.012 … 0.032]	0.034	0.030	[0.003 … 0.065]	0.029	0.004	[0.009 … 0.048]	0.025	0.235	[− 0.017 … 0.067]
Anterior region complete^b^	− 0.267	0.146	[− 0.629 … 0.094]	− 0.574	0.234	[− 1.523 … 0.374]	− 0.117	0.507	[-0.464 … 0.230]	− 0.548	0.228	[− 1.447 … 0.350]
	*R*^*2*^ = 0.017			*R*^2^ = 0.033			*R*^2^ = 0.030			*R*^2^ = 0.030		
Model 3												
Age^a^	0.009	0.403	[− 0.012 … 0.031]	0.029	0.058	[− 0.001 … 0.060]	0.029	0.004	[0.009 … 0.049]	0.019	0.346	[− 0.021 … 0.059]
Premolar region “sufficient”^b^	− 0.388	0.091	[− 0.838 … 0.062]	− 1.214	0.001	[− 1.942 … − 0.486]	− 0.007	0.977	[− 0.495 … 0.480]	− 1.617	< 0.001	[− 2.495 … − 0.738]
	*R*^*2*^ = 0.021			*R*^2^ = 0.076			*R*^2^ = 0.029			*R*^2^ = 0.142		
Model 4												
Age^a^	0.007	0.528	[− 0.015 … 0.029]	0.029	0.062	[− 0.001 … 0.059]	0.028	0.005	[0.009 … 0.048]	0.018	0.364	[-0.022 … 0.058]
Premolar region “sufficient”^b^	− 0.358	0.121	[− 0.812 … 0.096]	− 1.008	0.008	[− 1.749 … − 0.268]	0.049	0.844	[− 0.443 … 0.541]	− 1.626	< 0.001	[− 2.511 … − 0.741]
Molar region “sufficient”^b^	− 0.214	0.324	[− 0.642 … 0.213]	− 0.781	0.020	[− 1.436 … − 0.126]	− 0.342	0.126	[− 0.781 … 0.097]	0.210	0.743	[− 1.057 … 1.478]
	*R*^*2*^ = 0.026			*R*^*2*^ = 0.101			*R*^*2*^ = 0.037			*R*^*2*^ = 0.143		

*FDP*, fixed dental prosthesis; *PRDP*, partial removable dental prosthesis

^a^Per year

^b^Reference = no.

*95% confidence interval (CI)

### Masticatory ability and dental status based on natural teeth plus “teeth” replaced

Classified on the basis of natural teeth plus teeth replacement, mean number of “teeth” for participants with ≥ 10 “teeth” in each jaw was 27.25 ± 2.33, with 4.30 ± 3.03 natural posterior occluding pairs (Table 3). Mean MDS in this category was 2.05 ± 1.31. Participants with < 10 “teeth” in each jaw had a mean number of “teeth” of 14.74 ± 5.96, with 0.90 ± 1.32 natural posterior occluding pairs. In this category, mean MDS was 3.47 ± 1.47, again indicating worse masticatory ability for participants having < 10 “teeth” in each jaw.

The multiple regression analysis revealed a significant positive association between MDS and age for participants with ≥ 10 “teeth” in each jaw (Table 4: model 1: *P* = 0.004; *R*^2^ = 0.029). After adding the dentition variables “anterior region complete” (model 2; *R*^2^ = 0.030), “premolar region sufficient” (model 3, *R*^2^ = 0.029), “premolar region sufficient,” and “molar region sufficient” (model 4, *R*^2^ = 0.037) respectively to the models, age was still the only variable significantly associated with MDS. In contrast, for participants with < 10 “teeth” in each jaw, the models showed significant positive associations between MDS and “premolar region sufficient” both in models 3 (*P* < 0.001; *R*^2^ = 0.142) and 4 (*P* < 0.001; *R*^2^ = 0.143), indicating again the importance of a “sufficient” premolar region for the masticatory ability in this population.

### Effect of tooth replacement

The multiple regression analysis (Table 5) shows negative associations between MDS and the number of natural teeth as well as the number of “teeth” replaced by FDP or PRDP. For each additional tooth present or “tooth” added by FDP, the mean MDS decreased by 0.12 units, which indicate decreasing chewing difficulties. For each “tooth” added by PRDP, the MDS decreased by 0.06 units (*P* < 0.001; *R*^2^ = 0.278).

**Table 5:** Multiple regression model for assessing associations between mean masticatory disability score (MDS) and age, number of teeth, and number of “teeth” replaced by fixed dental prostheses (FDP) or by partial removable dental prostheses (PRDP) (*n* = 384)

	Effect	*P* value	95% CI*
Age^a^	0.010	0.265	[− 0.008 … 0.028]
Natural teeth^b^	− 0.116	< 0.001	[− 0.138 … − 0.095]
Teeth replaced by FDP^c^	− 0.121	< 0.001	[− 0.195 … − 0.047]
Teeth replaced by PRDP^c^	− 0.057	< 0.001	[− 0.081 … − 0.032]
*R*^*2*^ = 0.278			

^a^Per year

^b^Per natural tooth

^c^Per “tooth” replaced

*95% confidence interval (CI)

## Discussion

### Sample and the hierarchical dental functional classification system

This study investigated relationships between masticatory ability and age, and dental and prosthodontic status amongst a purposive sample of institutionalized older people living in eight nursing homes in Qingdao, China. This purposive sample might not be representative of all residents living in the participating eight nursing homes (e.g., people with indications of cognitive impairment were not invited to participate), nor of nursing homes and institutionalized elderly elsewhere in China. Nevertheless, we consider the outcomes to provide valuable information for oral health care providers and for authorities responsible for oral health care and its utilization.

For analyzing the relationship between masticatory ability and dental and prosthodontic status, the hierarchical dental functional classification system was used. As in adult general populations of previous studies, homogeneities after dichotomization of the groups of elders in the present study were moderate to good [33], which indicates that hierarchical dental functional classification system can be used not only for the general population but also for classifying dentitions of institutionalized older adults.

### Self-assessed masticatory function

For the assessment of masticatory ability, a wide variety of subjective methods (based on questionnaires) and objective methods (based on comminution and mixing ability tests) have been described in the dental literatures [34–36]. People’s satisfaction with their chewing ability is not determined entirely by their mechanical chewing function. Instead, it is a complex measure that embraces broad physical, social, and psychological components [37]. As subjective assessment of the masticatory process also includes the individual’s perception of aspects such as perceived comfort and pain, this patient-based assessment was considered most appropriate for evaluation of masticatory ability [38]. The use, as in the present study, of questionnaires that address difficulties with chewing different types of food is a common method for assessing masticatory ability. However, self-assessed masticatory ability and outcomes of functional tests correlate only weakly. In general, compared with the results of objective mastication tests, questionnaires that evaluate masticatory ability provide more optimistic outcomes, probably as a result of the capacity to adapt to an impaired dental situation [34].

Several studies on masticatory ability explicitly differentiate between soft and hard foods [39–42]. In the present study, differences in MDS for soft and hard foods were relatively small and varied from approximately 0.3 up to 1.2 (Table 3). These relatively small differences between chewing hard and soft foods may be due to the Chinese diet, which, unlike Western diets, contains few hard-fibrous foods, with most foods frequently eaten by Chinese people being steamed, fried, or boiled [43]. For this reason, we included uncooked foods, which were assigned to the category “hard foods.” Depending on the preparation, meat can be considered as soft or hard food. In Chinese cuisine, meat is mostly cooked and cut into small pieces and therefore considered as soft food [4]. In the present study, 12.3% of the participants found meat “very difficult to chew, avoiding this food,” while this percentage for raw carrots was 34.2.

### Age, dental conditions, and masticatory ability

In the present study, apart from dental and prosthodontic status, masticatory ability was analyzed in relation to the age of the participants. However, age in itself is not considered a risk factor for decreased masticatory ability, but mainly for two age-related factors: (maximum) bite force and saliva flow, both of which decrease with age [44]. Overall, the factor age was not associated with MDS (Table 5). In the category ≥ 10 natural teeth in each jaw, neither age nor dentition variables influenced MDS significantly: there were no or only minor chewing difficulties. However, in people with < 10 natural teeth in each jaw, age-related factors seemed to play a role but only if dental status was not considered. If dental status was considered in these people, “premolar region sufficient” and “molar region sufficient” decreased MDS significantly. In the category ≥ 10 teeth in each jaw, including tooth replacements, age-related factors influenced MDS significantly, even when dental status was considered. However, the models for this group explained only 3.7% of the variation in MDS. For people having < 10 teeth in each jaw, including “teeth” replaced, the premolar region influenced MDS significantly. It appeared that “premolar region sufficient” was significantly associated with MDS if the dental situation was “critical” as in the categories of people with < 10 teeth in each jaw, with or without teeth replacements.

Overall, number of natural teeth and number of teeth replaced by either FDP or PRDP influenced MDS significantly (Table 5). One of the main goals of rehabilitation of reduced dentitions is to restore impaired masticatory function by replacing missing teeth with FDPs or PRDPs. A systematic review showed that people with (extreme) shortened dental arches had reduced masticatory performance in the order of 30–40% and distal-extension RDPs compensated for this reduction only partially, in the order of 50% [20]. The results of the present study are in line with the results of that systematic review: “teeth” added by PRDP contributed only 50% of the masticatory ability of natural teeth or “teeth” added by FDP. The present study showed an effect on MDS for each FDP “tooth” added versus each RDP “tooth” added of − 0.121 and − 0.057 respectively (Table 5).

## Conclusions

In the institutionalized elderly, masticatory ability is significantly associated with the number of natural teeth as well as the number of teeth replaced by FDP or PRDP. In the present study, “premolar region sufficient” was significantly associated with masticatory ability if the dental situation was “critical” as in the categories of people having < 10 teeth in each jaw, with and without teeth replacements. “Teeth” added by PRDP contributed only 50% of the masticatory ability of natural teeth or “teeth” added by FDP.
